# Supplementary material for: Thermal Conductivity of Helium and Argon at High Pressure and High Temperature
Source: Materials (Basel). 2022 Sep 26;15(19):6681. doi: 10.3390/ma15196681 (PMC9573036; doi:10.3390/ma15196681)
Supplement: Supplementary file 1 [file materials-15-06681-s001.zip › materials-1927217-supplementary.pdf]

Supplementary Materials for

Thermal conductivity of helium and argon at high pressure and high temperature

Wen-Pin Hsieh [1,2,*], Yi-Chi, Tsao [1] and Chun-Hung Lin [1]

[1] *Institute of Earth Sciences, Academia Sinica, Nankang, Taipei 11529, Taiwan*

[2] *Department of Geosciences, National Taiwan University, Taipei 10617, Taiwan*

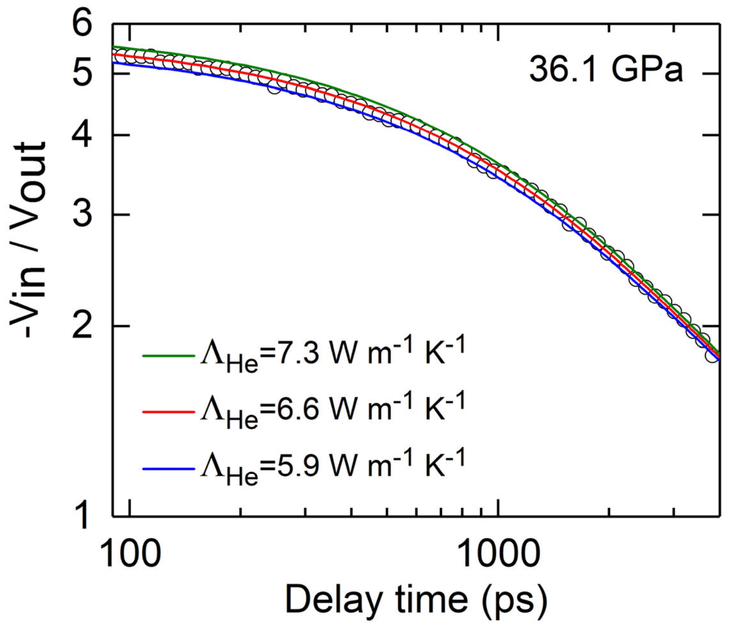

Figure S1. Representative TDTR data for the solid He at 36.1 GPa and room temperature. The temporal evolution of the ratio $-V_{in}/V_{out}$ (open circles) are fitted by the thermal model calculations (color curves). A thermal conductivity of Λ_{He}=6.6 W m^{-1} K^{-1} (red curve) gives a best-fit to the data using input parameters listed in Table S1. A testing 10% change in Λ (green and blue curves) causes the

model calculation deviating from the data. This indicates a high sensitivity of our thermal model fitting and a precisely derived Λ_{He} due to our high-quality data.

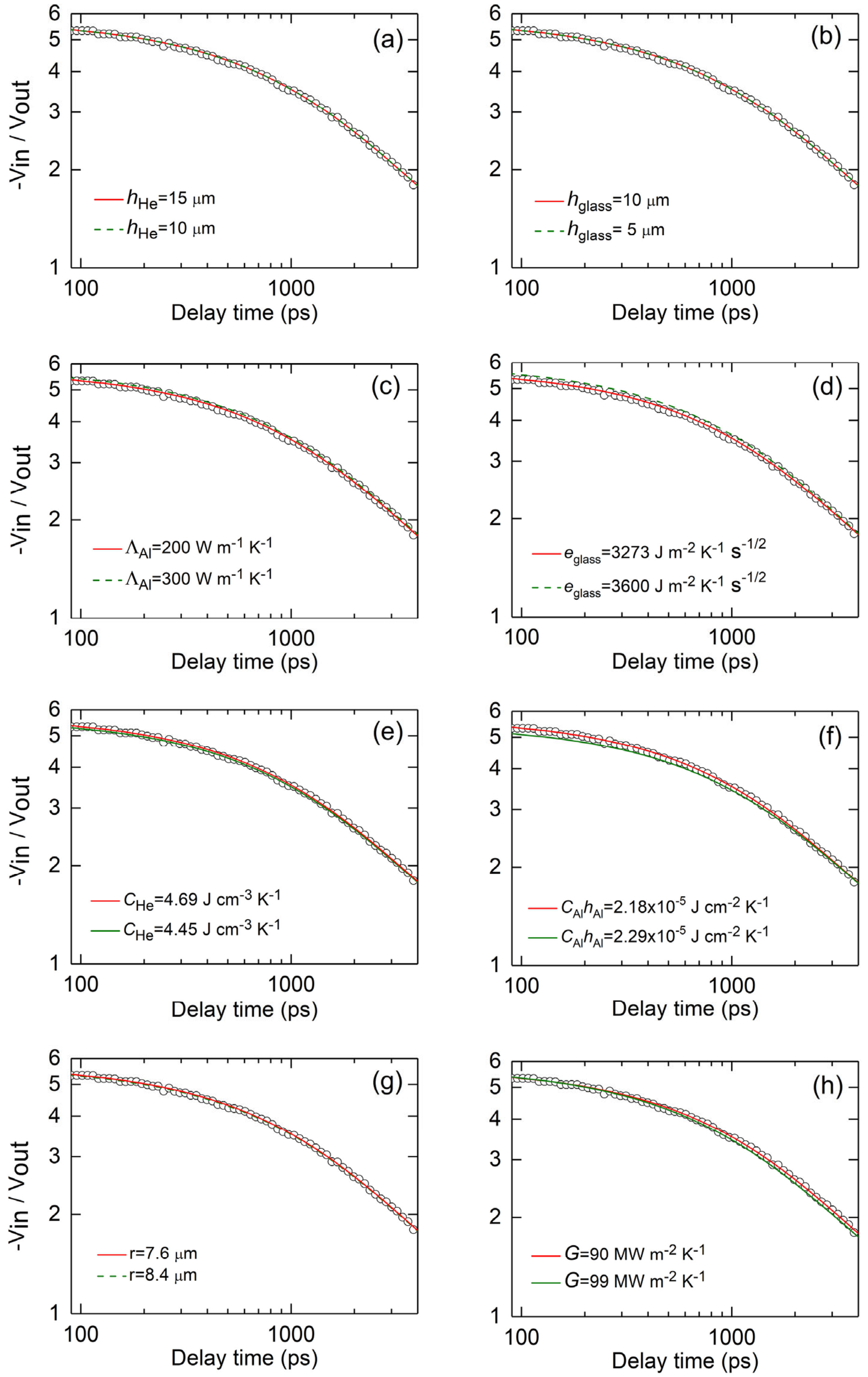

Figure S2. Sensitivity tests of the thermal model to each input parameter for the solid He at 36.1 GPa. As derived in Figure S1, here the thermal conductivity of He is fixed at 6.6 W m^{-1} K^{-1}. (a) and (b) The thermal model calculations remain the

same even when the thicknesses of He (h_{He}) and glass substrate (h_{glass}) change by 50%, respectively. Thus the uncertainties in the h_{He} and h_{glass} have no effects on the thermal conductivity of He (Λ_{He}). (c) The high thermal conductivity of Al film, Λ_{Al}, has a very minor influence on the Λ_{He}, even if a 50% change is applied. (d) If the thermal effusivity of the glass substrate, $e_{glass}=(\Lambda_{glass}C_{glass})^{1/2}$, is uncertain by 10%, it slightly shifts the model calculation, and requires Λ_{He} to decrease slightly from 6.6 to 6.2 W m^{-1} K^{-1} to fit the data again, i.e., translating ~6% error to the Λ_{He}. (e) Assuming the volumetric heat capacity of He, C_{He}, has 5% uncertainty, to fit the data the Λ_{He} needs to slightly increase to 6.8 W m^{-1} K^{-1}, ~3% uncertainty. (f) The major uncertainty source in our data analysis is from the uncertainty in Al heat capacity per unit area (product of its volumetric heat capacity and thickness, $C_{Al}h_{Al}$). Assuming a 5% uncertainty, it requires ~11% shift in the Λ_{He} to fit the data. (g) Even if the laser spot size is changed by 10%, it does not affect the model calculation. (h) A change in the thermal conductance of Al/He interface and Al/glass interface, G, by 10% slightly shifts the calculation, having very minor effect on the Λ_{He}.

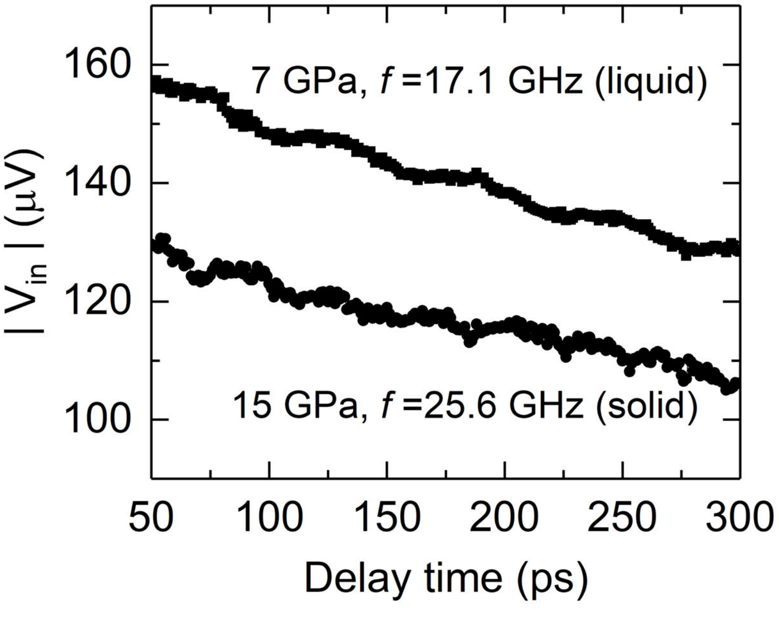

Figure S3. Representative time-domain stimulated Brillouin scattering spectra [1,2] for the liquid He at 7 GPa and solid He at 15 GPa and room temperature. The Brillouin frequency f at 7 GPa and 15 GPa was determined to be 17.1 GHz and 25.6 GHz, respectively, which were used to confirm the phase of the He [3].

Table S1. Input parameters for the bi-directional thermal model calculation at 36.1 GPa

P	C_{He}	C_{Al}	h_{Al}	$e_{glass} = (\Lambda_{glass} C_{glass})^{1/2}$	r	h_{He}/h_{glass}	Λ_{Al}	G
GPa	J cm^{-3} K^{-1}	J cm^{-3} K^{-1}	nm*	J m^{-2} K^{-1} s$^{-1/2}$	μm	μm	W m^{-1} K^{-1}	MW m^{-2} K^{-1}
36.1	4.69	2.67	81.6	3273	7.6	15/10	200	90

*In this experimental run, the Al thickness at ambient pressure is 88.6 nm measured by in situ picosecond acoustics. C_{He}: He heat capacity, C_{Al}: Al heat capacity [2], h_{Al}: Al thickness (calculated from Ref [4]), e_{glass}: glass thermal effusivity [1], r: laser spot size (measured in this study), h_{He}: He thickness (measured in this study), h_{glass}: glass thickness (measured in this study), Λ_{Al}: Al thermal conductivity [2], G: Thermal conductance of Al/He and Al/glass interfaces (measured in this study).

References

[1] W.-P. Hsieh, *High-Pressure Thermal Conductivity and Compressional Velocity of NaCl in B1 and B2 Phase*, Sci. Rep. **11**, 21321 (2021).

[2] W.-P. Hsieh, B. Chen, J. Li, P. Keblinski, and D. G. Cahill, *Pressure Tuning of the Thermal Conductivity of the Layered Muscovite Crystal*, Phys. Rev. B **80**, 180302 (2009).

[3] A. Polian and M. Grimsditch, *Elastic Properties and Density of Helium up to 20 Gpa*, Eur. Lett. **2**, 849 (1986).

[4] B. Chen, W.-P. Hsieh, D. G. Cahill, D. R. Trinkle, and J. Li, *Thermal Conductivity of Compressed H2O to 22 GPa: A Test of the Leibfried-Schlömann Equation*, Phys. Rev. B **83**, 132301 (2011).
